# Assessing the integration of social protection into infectious disease policies: a multi-country analysis of TB, HIV, and malaria strategies in Sub-Saharan Africa

**DOI:** 10.3389/fpubh.2026.1749640

**Published:** 2026-04-02

**Authors:** Isabelle Munyangaju, Dulce Osório, Edy Nacarapa

**Affiliations:** 1 Tinpswalo Research Association to Fight AIDS and TB, Chókwè, Mozambique; 2 Nucleo de Pesquisa Pediatria, Faculdade de Medicina, Universidade Eduardo Mondlane, Maputo, Mozambique

**Keywords:** health financing, HIV, malaria, policy analysis, social protection, Sub-Saharan Africa, tuberculosis, universal health coverage

## Abstract

**Background:**

Tuberculosis (TB), HIV, and malaria continue to impose major economic hardship on households in Sub-Saharan Africa. While global frameworks such as the WHO End TB Strategy and Universal Health Coverage (UHC) emphasize social protection, evidence remains limited on how national policies translate these commitments into practice.

**Objectives:**

This study assessed how national TB, HIV, and malaria policies and strategic plans in Kenya, Malawi, Mozambique, Nigeria, and Zambia define and operationalize social-protection mechanisms to reduce household economic burden. It also examined alignment with global targets on catastrophic-cost elimination and financial-risk protection, and identified policy–practice and equity gaps.

**Methods:**

A structured policy and document review was conducted in five countries, covering strategic plans, operational guidelines, and financing frameworks published between 2015 and 2025. Documents were sourced from government portals and partner repositories, including the Global Fund, PEPFAR, WHO IRIS, and UNAIDS. Analysis used a five-domain matrix adapted from WHO Health Systems Building Blocks and the Global UHC Readiness Framework: (A) Social Support Types, (B) Financial Protection, (C) Implementation Details, (D) Targeting & Equity, and (E) Coordination & Accountability. Domains were scored from 0 to 3 (0 = absent, 3 = high readiness). Two reviewers independently coded data and resolved discrepancies by consensus. Domain averages were used to generate national readiness indices for cross-country comparison.

**Results:**

A total of 111 policy documents were reviewed. No country had institutionalized catastrophic-cost monitoring. Overall readiness ranged from 2.6 to 2.9, indicating moderate to high preparedness to integrate social protection within infectious-disease programs. Malawi (2.9) and Zambia (2.8) scored highest due to costed, multisectoral frameworks and insurance linkages. Kenya (2.7) demonstrated strong coordination and legal anchoring but lacked formal catastrophic-cost monitoring. Mozambique (2.6) and Nigeria (2.7) scored lower in financial protection due to donor dependence and limited accountability. Implementation and coordination were strongest domains (3.0), while financial protection was weakest (2.1).

**Conclusion:**

Though national policies increasingly acknowledge social protection in infectious-disease control, significant gaps persist in financial-risk monitoring, budgeting, and accountability. Institutionalizing catastrophic-cost surveillance, integrating costed interventions into financing strategies, and reinforcing multisectoral coordination are critical to protect households from the economic impacts of infectious diseases.

## Introduction

Infectious diseases such as tuberculosis (TB), HIV, and malaria continue to exert a disproportionate social and economic burden on households across Sub-Saharan Africa (1). Despite global progress in reducing mortality and expanding access to preventive and treatment interventions, the indirect costs of illness such as lost income, transport expenses, and food insecurity remain significant barriers to care. For many low- and middle-income countries (LMICs), the economic consequences of infectious disease extend beyond health outcomes, deepening poverty, widening inequality, and perpetuating cycles of vulnerability among already disadvantaged populations (1–3).

Social protection has increasingly been recognized as a critical pillar in the global response to infectious diseases. Defined broadly as the set of public measures designed to provide income security, access to essential services, and protection against livelihood shocks. These mechanisms are central to achieving Universal Health Coverage (UHC) and the Sustainable Development Goals (SDGs 1.3 and 3.8) (4, 5). Within disease-specific frameworks, the World Health Organization (WHO) and partners have emphasized the need to eliminate catastrophic costs due to TB, and to strengthen equity and financial risk protection in HIV and malaria responses (1, 6). However, while these global commitments are well-articulated, translation into actionable national policies remains uneven, particularly in resource-constrained settings where health financing is fragmented and heavily donor-dependent (7–9).

Across Sub-Saharan Africa, the intersection of health policy and social protection is increasingly recognized but rarely harmonized. In many settings, TB and HIV programs operate vertical assistance schemes (e.g., nutrition packages, transport vouchers, or conditional cash transfers), yet these are often pilot-scale, donor-funded, or poorly integrated into broader national social protection systems (10, 11). Similarly, malaria control programs may provide preventive commodities free of charge but lack mechanisms to cushion households from income loss due to illness (12, 13). These fragmented approaches reveal persistent policy-practice gaps and highlight the need for an integrated understanding of how social protection is conceptualized, financed, and implemented within national health frameworks (14–17).

This study addresses this gap by systematically reviewing national policy and strategic documents related to TB, HIV, and malaria across Kenya, Malawi, Mozambique, Zambia, and Nigeria, countries that together represent diverse epidemiological, economic, and governance contexts in Sub-Saharan Africa. Through this analysis, the study seeks to provide evidence on the extent and quality of social protection integration within infectious disease policies, assess alignment with global targets on financial-risk protection, and inform multisectoral policy dialogue on strengthening health system resilience through social protection integration.

In this study, social protection is defined as structured public measures such as cash or in-kind transfers, social insurance, fee waivers, and livelihood-support mechanisms, designed to reduce income loss, prevent catastrophic expenditure, and protect households from economic shocks associated with illness (18–20). This definition aligns with ILO and World Bank frameworks that conceptualize social protection as income security and risk mitigation across the life course, while in the health sector specifically emphasizing financial-risk protection and mitigation of direct and indirect costs of illness.

Importantly, this study assesses social protection as it is conceptualized and operationalized within health-sector policy documents. It does not evaluate broader national welfare systems beyond the health-policy interface.

## Objectives

- Primary Objective: To identify and compare how national TB, HIV, and malaria policies and strategic plans define and operationalize social protection measures to mitigate household economic burden.
- Secondary Objectives: (i) To assess the alignment of national policy frameworks with global targets such as the WHO End TB Strategy and UHC commitments; and (ii) To identify policy-practice and equity gaps, including the extent to which gender, poverty, and rurality considerations are incorporated into implementation strategies.

## Methods

### Study design and scope

This study employed a structured policy and document review to examine how national TB, HIV, and malaria strategies in Kenya, Malawi, Mozambique, Zambia, and Nigeria define and operationalize social protection mechanisms to mitigate household economic burden from infectious diseases. The five countries were purposively selected to capture variation in epidemiological burden (high TB/HIV vs. mixed malaria burden), levels of health-system decentralization, and stages of UHC reform, as well as differences in domestic fiscal capacity and donor dependency. Together, these countries represent diverse governance and financing models within Sub-Saharan Africa, allowing comparative policy analysis across contrasting contexts.

Documents were sourced from official government portals (Ministries of Health, Finance, and Social Protection), and from partner repositories such as the Global Fund, PEPFAR, WHO IRIS, and UNAIDS. The search covered policies published from 2015 to the present. Only final or officially endorsed versions were included to ensure policy relevance and alignment with national strategic directions.

### Search strategy

A structured search strategy was applied across all platforms and for each country using combinations of the following terms: “national strategic plan,” “TB/HIV/Malaria strategy,” “social protection,” “community support,” “financial risk protection,” “patient support package,” “health financing,” and “vulnerability mitigation.” Boolean operators (AND/OR) were used to refine results. Snowballing was done by screening bibliographies and cross-referencing documents cited within national strategies. At times documents mentioned in the national strategies were not available online, in these cases contact was made with these countries to get the documents.

Documents were included if they met all the following criteria:

- If the documents were national strategic plans, operational guidelines, monitoring and evaluation frameworks, health-financing strategies, investment cases, or policy briefs with explicit reference to social protection, patient support, community-based support, or financial-risk protection. If they addressed at least one of the following domains:
- Social protection mechanisms (cash transfers, transport vouchers, nutritional support, disability grants, community-based support packages)
- Financial risk protection or mitigation of catastrophic health costs
- Integration or alignment of social protection within TB/HIV/malaria programming
- Linkages to national social protection systems or social welfare programs
- If they were national-level documents applicable to the whole health system or specific disease programs.
- If they were published from 2015 onwards.
- No language restrictions were applied during the search; however, the majority of official policy documents in the selected countries were available in English (Kenya, Malawi, Zambia, and Nigeria) or Portuguese (Mozambique). Portuguese documents were reviewed in the original language.

Documents were excluded if they were primarily clinical guidelines lacking socioeconomic or health-financing content; and if they were pure academic publications without direct policy relevance.

### Conceptual foundation

The review was grounded in the principles of Universal Health Coverage (UHC) and the Sustainable Development Goals (SDGs 1.3 and 3.8) (21–24), emphasizing the intersection of health financing, equity, and social protection. It examined the extent to which countries align their infectious disease strategies with the goal of eliminating catastrophic health expenditures and promoting financial risk protection.

### Analytical framework

**Figure 1:**
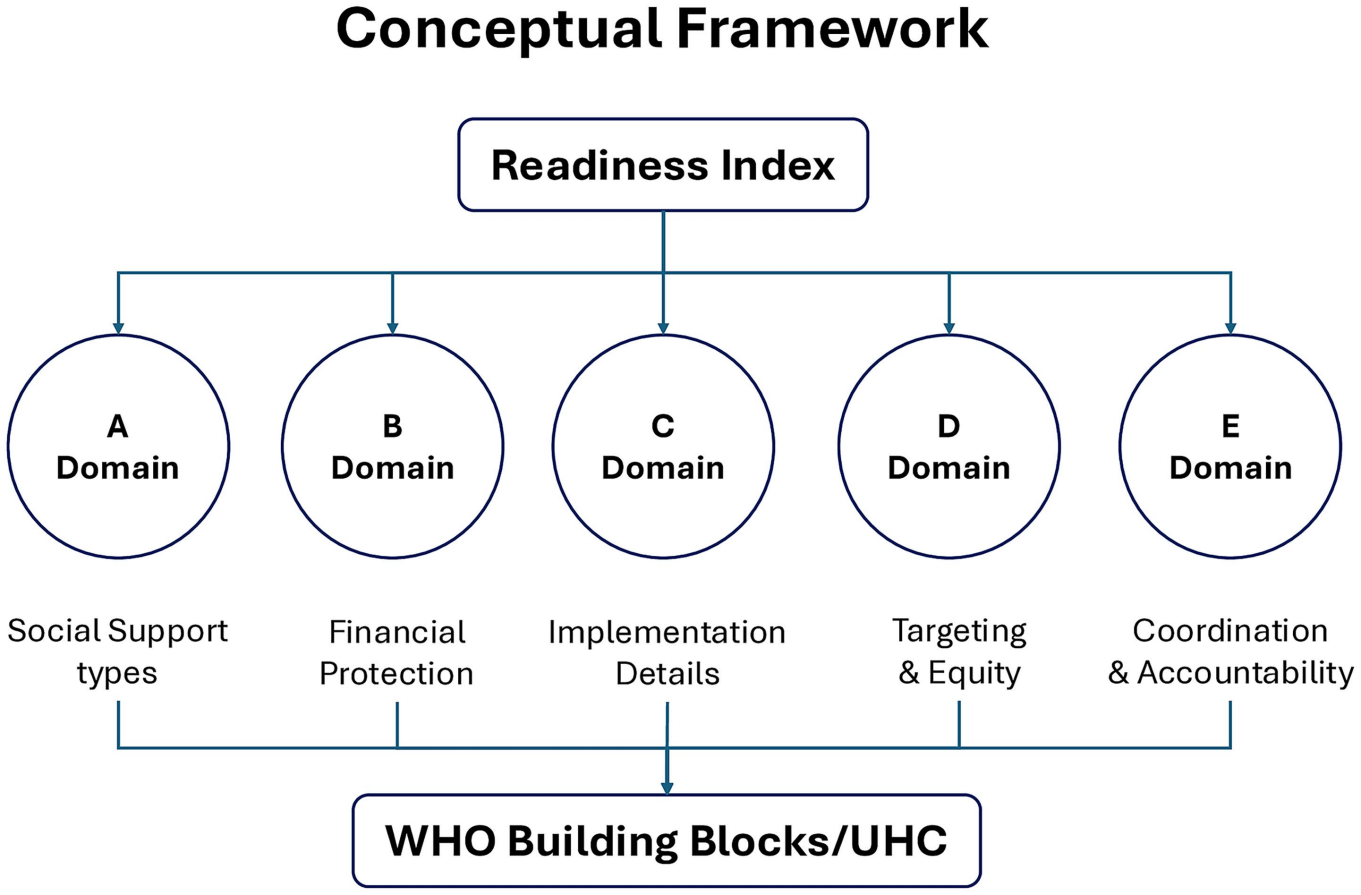
A flowchart of the foundation of the WHO building blocks/UHC, leading to the five domains (A–E), and results in the readiness index.

The analysis was guided by a five-domain coding matrix, adapted from the World Health Organization’s Health Systems Building Blocks and the Global UHC Readiness Framework (25–27). The five domains were (Figure 1):

- Domain A—Social Support Types: Evaluated the extent of social protection, community participation, and patient-centred support mechanisms in TB, HIV, and malaria care.
- Domain B—Financial Protection: Assessed whether national plans ensure access to free or subsidized services and include monitoring of household catastrophic costs.
- Domain C—Implementation Details: Reviewed the presence of budget lines, funding sources, and responsible agencies for execution and monitoring.
- Domain D—Targeting & Equity: Analysed inclusion of vulnerable and high-risk groups (e.g., miners, women, children, rural populations) and the use of equity-driven strategies.
- Domain E—Coordination & Accountability: Examined legal frameworks, multisectoral coordination, and accountability mechanisms for oversight and feedback.

Each domain was scored using a three-point ordinal scale (0–3): 0 = Absent; 1 = Weak; 2 = Moderate; 3 = High readiness. All domains were weighted equally. This scoring approach, adapted from WHO policy-readiness frameworks, enabled systematic comparison of policy intent and operational maturity across the five study countries.

All domains were weighted equally to avoid imposing normative assumptions about the relative importance of individual components of social protection. The objective of the scoring system was comparative policy-readiness assessment rather than impact measurement. We acknowledge that alternative weighting strategies particularly those emphasizing financial-risk protection could yield different rankings; this is discussed in the limitations.

### Data extraction and coding

Textual content from each policy document was systematically reviewed using a deductive coding approach based on the five domains. For each domain, relevant passages were identified, extracted verbatim, and tabulated with their page references, evidence quotes, and interpretive notes.

Two reviewers independently coded all documents using a standardized Excel matrix developed for the study. Coding discrepancies were discussed and resolved through consensus. A traffic-light colour scheme (red = low, yellow = moderate, green = high readiness) was applied to visualize cross-country patterns and domain-level variation.

### Scoring and interpretation

Domain-specific scores were averaged to calculate an overall readiness index (0–3) for each document and country. Interpretation thresholds were (Table 1):

**Table 1:** Scoring system (0–3 scale).

Score	Domain A: Social Support Types	Domain B: Financial Protection	Domain C: Implementation Details	Domain D: Targeting & Equity	Domain E: Coordination & Accountability
3 (High)	Explicit mention of free services + structured social support (cash/in-kind) + community engagement mechanisms	Free services + functional insurance/waiver system + defined catastrophic-cost monitoring	Costed multi-year plan + clear funding sources + designated implementing agency	Explicit inclusion of vulnerable groups + measurable equity indicators + budget allocation	Legal framework + multisectoral coordination + grievance/feedback system
2 (Moderate)	Free services + some social support (e.g., transport vouchers) but limited scale-up	Free services + some financial protection (insurance) but no cost monitoring	Implementation plan present but partially costed or donor-dependent	Vulnerable groups mentioned but no measurable indicators or budget	Coordination structures exist but weak accountability mechanisms
1 (Weak)	Vague commitment to social support or pilot programs only	Vague commitment to reducing costs or free services mentioned but not guaranteed	Implementation details unclear or unfunded	Equity mentioned rhetorically without operationalization	Coordination mentioned but no formal structure
0 (Absent)	No mention of social support	No mention of financial protection	No implementation details	No mention of equity or targeting	No mention of coordination or accountability

- 0.0–1.0 = Low readiness (policy intent absent or minimal),
- 1.1–2.0 = Moderate readiness (policy intent present but weakly operationalized),
- 2.1–3.0 = High readiness (policy well-defined, costed, and supported by accountability mechanisms).

The composite index then provided a standardized measure of policy preparedness for mitigating household economic burden through social protection measures in infectious disease programs.

### Ethical considerations

This research involved secondary analysis of publicly available documents and did not include human subjects or identifiable patient data. As such, formal ethics approval was not required. All materials were handled and cited according to academic and institutional standards for research integrity and transparency.

## Results

A total of 111 policy and strategic documents were reviewed across five countries: Kenya (27), Malawi (17), Mozambique (25), Nigeria (17), and Zambia (25). The corpus comprised a diverse range of national-level materials, including 42 national strategic plans, 31 guidelines, 15 policy papers, 9 reports, 8 peer-reviewed articles, 1 operational plan, 1 case study, 1 concept note, 1 flyer, 1 issue paper, and 1 study protocol related TB, HIV, malaria, and integrated health programs published between 2015 and 20205 (Supplementary material 1).

### Overall policy readiness: high scores mask a critical gap in financial protection

**Figure 2:**
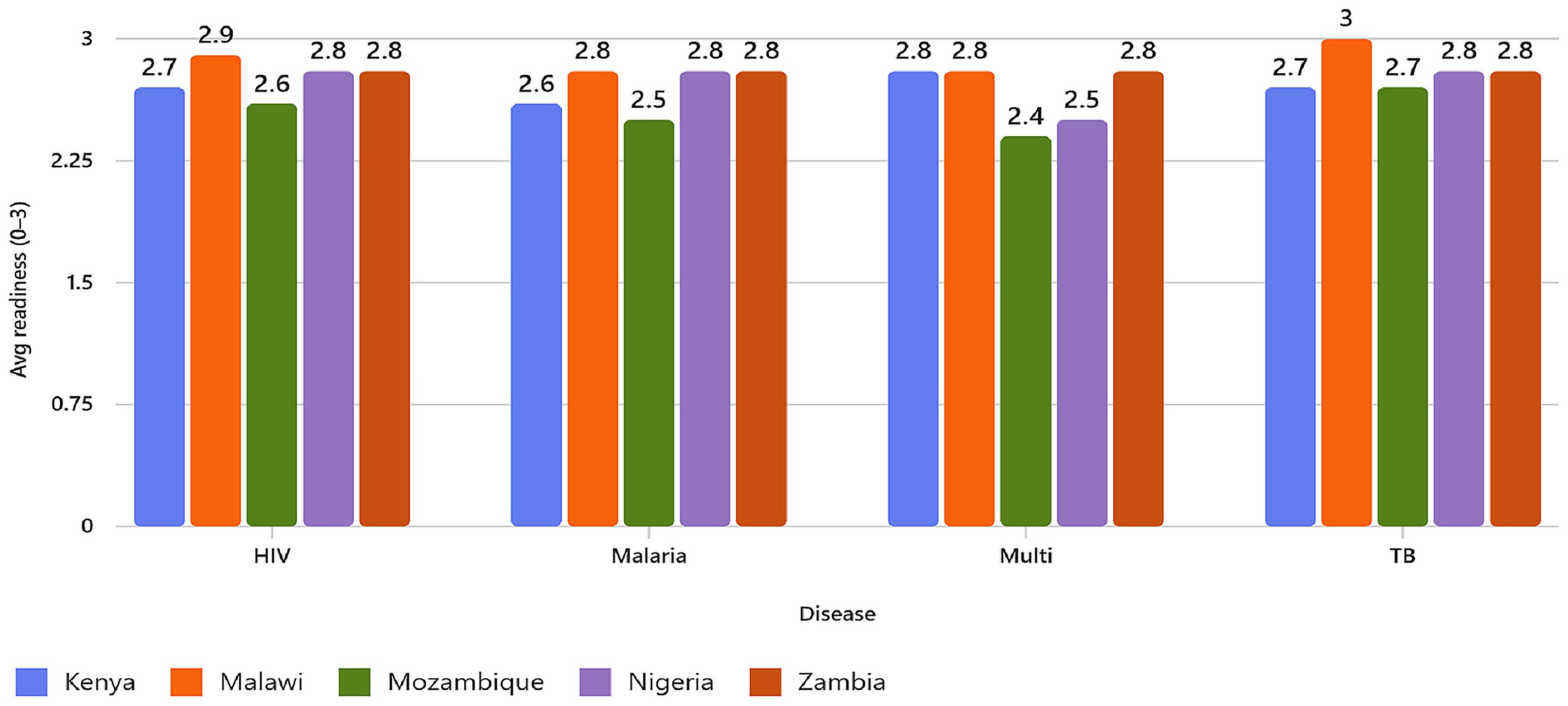
Average readiness by country and disease: Grouped bar chart comparing readiness scores across TB, HIV, malaria, and multi-disease strategies in five countries.

Across all countries and disease programs, the average readiness index ranged from 2.4 to 3.0, indicating moderate to high policy preparedness for integrating social protection measures within infectious diseases responses (Figure 1). Zambia, Malawi, and Kenya demonstrated the strongest readiness (average 2.8–3.0), anchored by costed multi-year strategic plans, legal frameworks, and multisectoral governance structures, and efforts to integrate health financing within national insurance mechanisms. Mozambique and Nigeria scored slightly lower (2.4–2.8), mainly due to gaps in financial-risk monitoring, grievance mechanisms, and limited domestic financing (Figure 2).

Notably, the composite readiness index may obscure critical domain-level disparities. While overall scores suggest moderate-to-high readiness, Financial Protection (Domain B) consistently scored lowest across countries. The high composite scores are driven largely by strong implementation and coordination structures rather than robust financial-risk protection mechanisms. Thus, “high readiness” should therefore be interpreted as institutional preparedness within health-sector frameworks rather than comprehensive social protection in the broader welfare-state sense.

### Cross-country patterns in policy readiness

Kenya showed high readiness (2.6–2.9) across disease programs, supported by strong legal frameworks, alignment with UHC, Vision 2030 (28), and SDG 3.8. Its TB and multi-disease plans displayed strong decentralization, multisectoral coordination, including community participation. However, explicit financial-risk protection metrics, grievance systems, and cash-support mechanisms remain underdeveloped.

Malawi achieved the highest overall readiness (up to 3.0 for TB and 2.9 for HIV), reflecting solid institutional alignment with UHC and Malawi Vision 2063 (29), costed implementation plans, and integration of donor and domestic financing under the “One Plan-One Budget-One M&E” principle. However, this high score is driven primarily by robust governance, coordination, and implementation structures rather than by institutionalized income-support or catastrophic-cost monitoring mechanisms. Direct household-level cash assistance, formal insurance coverage, and systematic economic-burden tracking remain largely absent. Under a narrower definition of social protection centred on income replacement and financial-risk monitoring, Malawi’s readiness would be more modest.

Mozambique recorded moderate readiness (2.4–2.7). National frameworks such as PEN V (HIV), PESS, and the Malaria and TB Strategic Plans demonstrate strong equity intent and multisectoral collaboration. However, implementation is hindered by donor reliance, fiscal constraints, and the weakest financial-protection instruments among the five countries.

Nigeria also showed moderate readiness (2.5–2.8), particularly for HIV and TB programs, emphasizing PHC revitalization, gender inclusion, and integration with social protection systems. Despite strong design and coordination mechanisms through institutions like National AIDS, Sexually Transmitted Infections Control and Hepatitis Programme (NASCP), The National Tuberculosis and Leprosy Control Programme (NTBLCP), and National Health Insurance (NHIS), household-level economic protection and grievance systems remain underdeveloped, and implementation was hindered by persistent donor dependence.

**Figure 3:**
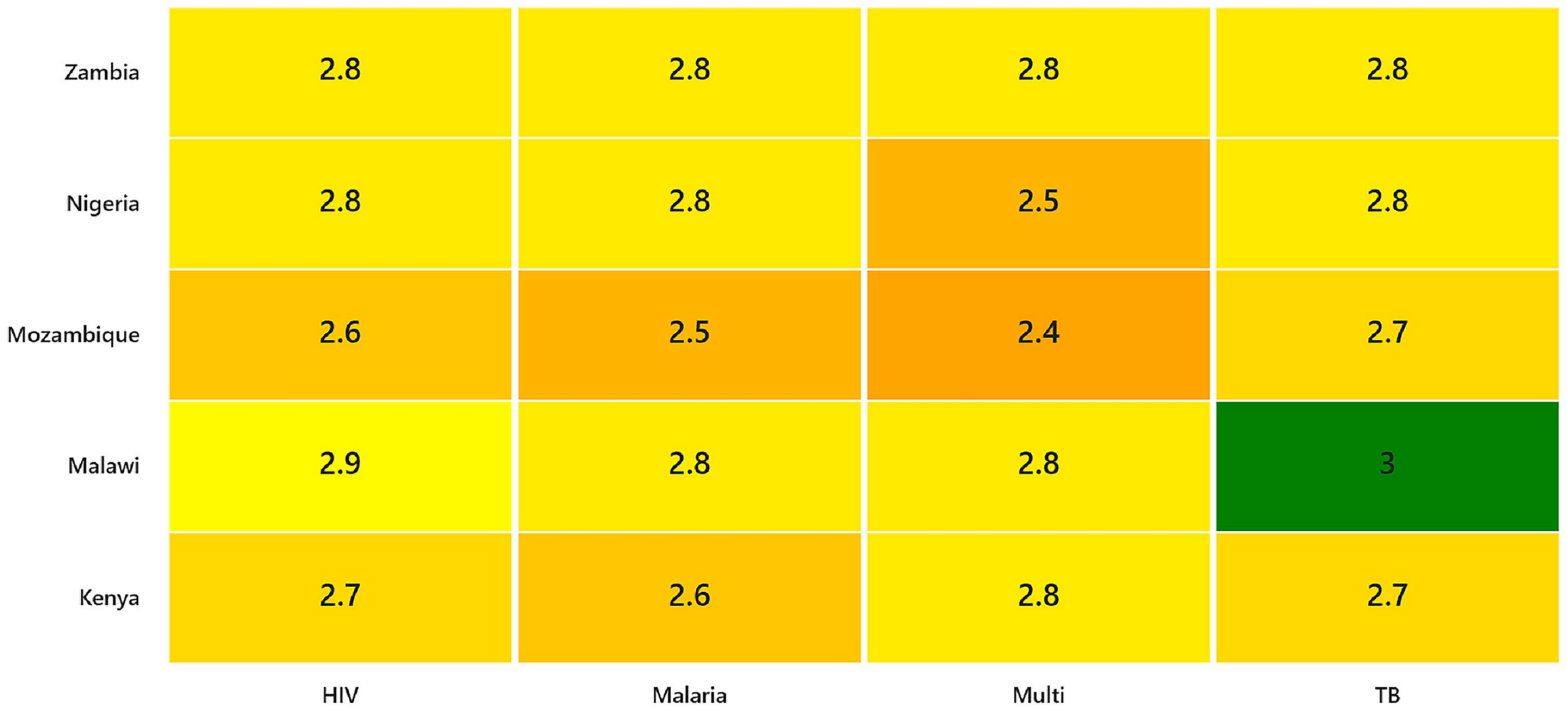
Heatmap of policy readiness scores: Traffic-light colour scheme [green = high readiness (>2.7), yellow = moderate readiness (2.6–2.7), orange = low readiness (<2.6)] applied to visualize readiness across countries and diseases.

Zambia scored consistently high (2.8) across all programs. The integration of NHIMA, cash-transfer programs, and fiscal-space reforms under Vision 2030 has strengthened accountability, service coverage, and rights-based inclusion. Persistent challenges include limited catastrophic-cost monitoring and sustainability risks from donor co-financing (Table 2 and Figure 3).

**Table 2:** Cross-country synthesis of policy readiness for social protection integration (2015–2025).

Country	Disease area(s)	# Docs reviewed	Average readiness (0–3)	Key strengths	Key gaps/challenges
Kenya	TB/HIV/malaria/multi-disease	27	2.8–2.9	• Strong legal frameworks and UHC alignment (Vision 2030). • Multisectoral coordination committees functioning across TB and multi-disease plans. • Community engagement and gender-responsive programming.	• No routine catastrophic-cost monitoring. • Weak grievance and feedback systems. • Limited cash or in-kind support institutionalization.
Malawi	TB/HIV/malaria/health financing	17	2.8–3.0	• Strong integration of donor + domestic funding under “One Plan–One Budget–One M&E.” • Explicit social-support components (transport vouchers, food packages). • High alignment with UHC and Vision 2063.	• No catastrophic-cost indicators. • Donor dependency and limited domestic fiscal space. • Weak grievance-redress mechanisms.
Mozambique	TB/HIV/malaria/health sector	25	2.4–2.7	• Legal anchoring through PESS 2020–2029 and PEN V. • Equity focus (gender, community, and rural inclusion). • Active multisectoral partnerships.	• Absence of financial-risk or catastrophic-cost monitoring. • High donor reliance; limited domestic funding. • Weak grievance and citizen-feedback systems.
Nigeria	TB/HIV/malaria/PHC systems	17	2.5–2.8	• Emphasis on PHC revitalization and social-protection linkages. • Gender-equity policies within HIV and TB plans. • NHIS and BHCPF frameworks provide financial-protection entry points.	• Limited household-cost monitoring and data systems. • Fragmented accountability and grievance structures. • Persistent donor dependence and implementation bottlenecks.
Zambia	TB/HIV/malaria/multi-disease	25	≈ 2.8	• Integration of NHIMA and cash-transfer schemes within national UHC agenda. • Costed, multi-year strategies with decentralized PHC delivery. • Strong interministerial collaboration.	• Absence of catastrophic-cost tracking. • Weak grievance-redress and citizen-feedback mechanisms. • Sustainability risks from donor co-financing.

#### Domain-level analysis

*Social Support Types (Domain A)*: All countries guarantee free diagnosis and treatment for TB, HIV, and malaria. Kenya and Malawi integrate community outreach, peer support, and gender-responsive programming. Mozambique and Nigeria rely more on donor-funded pilots, while Zambia has begun formal integration with social-assistance systems. *Financial Protection (Domain B)*: Although free ART and TB treatment are standard, systematic monitoring of catastrophic costs is absent across all five countries. Health insurance schemes in Kenya, Nigeria, and Zambia show promise but remain limited in population coverage. *Implementation Details (Domain C)*: Budget lines for social protection are inconsistently defined. Malawi and Zambia have costed, multi-year frameworks, while Kenya shows partial costing and Mozambique and Nigeria rely on donor-linked budgets. *Targeting & Equity (Domain D)*: Gender, youth, and rural equity considerations are explicitly included in HIV and TB plans across all countries. However, few frameworks operationalize these priorities through measurable indicators or routine monitoring. *Coordination & Accountability (Domain E)*: All five countries maintain multisectoral coordination structures, including interministerial committees and partner platforms. Yet formal grievance-redress mechanisms and citizen-feedback systems remain the weakest elements across contexts (Tables 3A,B, 4).

**Table 3:** Domain-level readiness summary across five countries.

A	
Domains	Readiness assessment
Strongest Domains: Implementation, Equity, and Coordination (Domains C, D, E)	The highest scores were consistently observed in Domain C (Implementation Details), Domain D (Targeting & Equity), and Domain E (Coordination & Accountability), with averages between 2.9 and 3.0. All countries maintained multisectoral coordination structures and implementation oversight by national disease programs. Furthermore, gender, youth, and rural equity considerations were explicitly referenced in most policy frameworks.
Weakest Domain: Financial Protection (Domain B)	In stark contrast, Domain B (Financial Protection) was the weakest area for all countries, with scores ranging from 1.7 to 2.7. Although free antiretroviral therapy (ART) and TB treatment are standard, *not a single country had institutionalized systematic monitoring of catastrophic health expenditures*—a core indicator for the WHO End TB Strategy and Universal Health Coverage (UHC). Health insurance schemes (e.g., Kenya’s NHIF, Zambia’s NHIMA) showed promise but offered limited population coverage.
Social Support (Domain A)	All countries guaranteed free diagnosis and treatment. Social support types such as transport vouchers and food packages were present but often fragmented, pilot-scale, and reliant on donor funding, particularly in Mozambique and Nigeria.

B				
Domain	Description	Cross-country findings (Kenya, Malawi, Mozambique, Nigeria, Zambia)	Average score range (0–3)	Overall interpretation
A. Social Support Types	Extent of patient-centred and community-based social protection (cash, in-kind, or psychosocial support).	• All countries guarantee free diagnosis and treatment for TB, HIV, and malaria. • Kenya and Malawi include transport vouchers, food packages, and community adherence groups. • Zambia links community outreach with existing social-assistance schemes. • Mozambique and Nigeria rely mainly on donor-driven pilots with limited scale-up.	2.7–2.9	Social-support mechanisms exist but remain fragmented and unsustainably financed.
B. Financial Protection	Presence of insurance, waivers, and catastrophic-cost monitoring mechanisms.	• Free ART and TB treatment across all countries. • Kenya (NHIF), Zambia (NHIMA), and Nigeria (NHIS/BHCPF) provide partial coverage but with limited reach. • No country has institutionalized WHO catastrophic-cost indicators. • Financing largely donor-dependent.	1.7–2.7	Universal free services achieved but formal financial-risk monitoring is absent.
C. Implementation Details	Presence of budget lines, funding sources, and institutional responsibilities.	• Malawi and Zambia have costed multi-year frameworks. • Kenya has partial costing through UHC reforms. • Mozambique and Nigeria depend on external grants with minimal domestic allocation. • Implementation oversight by national disease programs in all settings.	2.9–3.0	Implementation frameworks exist but unevenly costed and reliant on partners.
D. Targeting & Equity	Inclusion of vulnerable groups (gender, youth, rurality, informal workers) and use of equity-driven strategies.	• Gender and rural inclusion explicitly referenced in HIV and TB plans across all countries. • Equity rarely linked to measurable indicators or budget allocations. • Malawi and Kenya perform best in mainstreaming gender and adolescent programming.	2.9–3.0	Strong policy rhetoric on equity but weak operationalization and measurement.
E. Coordination & Accountability	Legal frameworks, multisectoral coordination, and grievance or feedback systems.	• All five countries maintain interministerial or partner coordination structures. • Kenya and Zambia have functional technical working groups aligned with UHC governance. • Formal grievance-redress or citizen-feedback systems are absent or nascent in all contexts.	2.9–3.0	Coordination mechanisms are robust; accountability and grievance systems remain weak.

**Table 4:** Domain-level readiness scores across five countries (2015–2025).

Country	A = Social Support	B = Financial Protection	C = Implementation Details	D = Targeting & Equity	E = Coordination & Accountability
Kenya	2.7	2.1	3.0	2.9	3.0
Malawi	2.7	2.7	3.0	3.0	3.0
Mozambique	2.4	1.7	3.0	2.8	3.0
Nigeria	2.8	2.0	2.9	3.0	2.9
Zambia	2.9	2.2	3.0	3.0	3.0

## Discussion

This study set out to identify and compare how national TB, HIV, and malaria policies in five Sub-Saharan African countries define and operationalize social protection to mitigate household economic burden. By applying a structured five-domain analytical framework, the findings show that while social protection is increasingly referenced in policy rhetoric, its operationalization remains uneven and primarily concentrated in governance, coordination, and implementation structures rather than in measurable financial-risk protection mechanisms. In other words, policy readiness reflects institutional preparedness within the health sector, but does not consistently translate into comprehensive income protection or systematic monitoring of catastrophic costs. These findings reveal important gaps between global commitments to eliminate economic hardship due to infectious diseases and the practical policy instruments currently embedded within national frameworks (Figure 3).

These findings align with growing evidence from the region, where limited progress has been made in translating social protection commitments into sustainable, nationally financed interventions. Consistent with analyses by the WHO and The Global Fund, our results show that while TB and HIV programs frequently incorporate patient-centred support (e.g., nutritional supplementation, transport vouchers, or community follow-up), these interventions remain donor-funded and weakly embedded in national financing systems (6, 30). This fragmentation reflects what Boccia et al. (31) termed parallel social protection, isolated initiatives that fail to link with broader welfare mechanisms (32).

The absence of catastrophic-cost monitoring, a core WHO End TB indicator, across all five countries further illustrates this gap between policy aspiration and measurable accountability. This finding echoes results from the Global Tuberculosis Report (33) and WHO’s TB Patient Cost Surveys, which highlight limited institutionalization of economic burden surveillance in Africa (34). Despite explicit commitments to eliminate catastrophic costs by 2020, none of the reviewed national plans had adopted formal measurement frameworks. These results parallel conclusions from Barasa et al. (31) in Kenya and Wagstaff et al. (35), who noted that financial-risk protection indicators remain underdeveloped within LMIC health financing strategies (36).

A similar disconnect is evident in HIV policy implementation. Although recent UNAIDS (2021) and International Labour Organization (ILO) (2020) reviews indicate that HIV strategies frequently acknowledge social protection and gender inclusion, these components are rarely translated into measurable or costed interventions. Policy rhetoric often invokes inclusion of women, adolescents, or key populations, yet implementation frameworks seldom translate such commitments into targeted actions or dedicated budgets. This pattern of policy intent without fiscal backing reflects a wider governance challenge identified in UHC readiness assessments conducted by WHO and the World Bank (37–39).

Compared with TB and HIV programs, malaria strategies demonstrate relatively weaker integration of social protection. Our domain-level analysis showed moderate readiness (2.4–2.7) for malaria frameworks, consistent with findings from the World Malaria Report (40) and evaluations by the Roll Back Malaria (RBM) Partnership (41). Malaria control efforts remain predominantly biomedical, focusing on prevention and case management while offering limited provisions for household-level economic resilience. This imbalance underscores the need to broaden malaria responses beyond clinical interventions to include mechanisms that mitigate income loss and financial vulnerability during illness.

Across the five countries, readiness was highest in Malawi (2.9) and Zambia (2.8), where costed multi-year strategies and national insurance mechanisms (National Health Insurance Management Authority (NHIMA), social-cash-transfer schemes) are embedded within UHC reforms. Kenya also demonstrated strong institutional readiness (2.7), anchored in Vision 2030 and the National Hospital Insurance Fund (NHIF) (42–44). Conversely, Mozambique (2.6) and Nigeria exhibited weaker financial-protection scores, (2.6) and (2.7) respectively, and higher donor dependence, mirroring patterns described in multi-country UHC tracking reports (39).

Comparative evidence from other regions offers instructive contrasts. In Latin America, countries such as Brazil and Peru have institutionalized TB social protection through conditional cash transfers and employment reintegration schemes linked to national poverty-alleviation programs. Evaluations of Brazil’s *Bolsa Família* and Peru’s *JUNTOS* initiatives demonstrate measurable improvements in treatment adherence and reduced household financial distress (45, 46). Similarly, in Asia, programs in India and the Philippines have piloted TB-specific cash benefits integrated into national health insurance platforms (e.g., *Nikshay Poshan Yojana*), illustrating how policy coherence and fiscal mainstreaming can enhance sustainability (47, 48). These examples highlight that successful and sustained social-protection integration depends on domestic policy ownership, fiscal commitment, and intersectoral coordination; factors that remain limited in the Sub-Saharan African context.

Social protection is inherently multisectoral, extending beyond ministries of health to include social welfare, finance, labour, and broader redistributive fiscal policy (49, 50). Evaluating social protection through a health-sector framework therefore presents both opportunity and limitation. On the one hand, infectious-disease programmes often possess stronger institutional structures, clearer monitoring systems, and greater funding flows than other social sectors, positioning them as important entry points for economic protection interventions (51, 52). On the other hand, health systems alone cannot substitute for comprehensive welfare systems that provide income replacement, unemployment protection, or long-term livelihood security (50).

The WHO framework applied in this study captures coordination and multisectoral intent, but it does not fully measure the redistributive and income-security functions central to conventional definitions of social protection. As such, the readiness scores should be interpreted as reflecting the capacity of health-sector policies to support social protection, rather than the overall strength of national social protection systems. This distinction is critical when interpreting high composite scores alongside persistent weaknesses in financial-risk monitoring and income-support mechanisms.

Based on the identified gaps and comparative insights, four strategic priorities are recommended to strengthen the integration of social protection within infectious disease policies. First, all countries should institutionalize WHO-recommended catastrophic-cost monitoring across TB, HIV, and malaria programs to generate empirical evidence on household economic burden. Second, legal and financing frameworks must evolve from aspirational commitments to costed, budgeted interventions embedded within national health-financing strategies and social insurance schemes such as NHIF and NHIMA to ensure sustainability. Third, equity dimensions (gender, poverty, and rurality) should be operationalized through measurable indicators and linked to accountability systems that enable citizen feedback and grievance redress. Finally, multisectoral coordination between the Ministries of Health, Finance, and Social Protection should be strengthened through joint planning platforms to harmonize resources and align UHC reforms, drawing lessons from countries like Brazil, India, and Kenya where interministerial collaboration has strengthened policy coherence and health-system resilience.

## Strengths and limitations

This study offers a novel and systematic assessment of the integration of social protection within infectious disease programs in diverse Sub-Saharan countries, addressing a gap in regional policy analysis. By applying a standardized five-domain analytical framework and scoring system, it provides a structured and replicable approach to evaluating policy readiness across diverse national contexts. The inclusion of five countries with varying epidemiological and economic profiles enhances the regional relevance and transferability of findings.

Nevertheless, the study has several limitations. First, it relied solely on policy document analysis, which captures intent rather than implementation performance. Consequently, readiness scores may not reflect on-the-ground performance. Second, the use of three-point ordinal scale, while transparent and replicable, introduces subjective judgement in interpreting textual evidence. Third, subnational and purely programmatic guidelines were excluded, which in highly decentralized systems like Kenya and Nigeria, these may contain more operational details. Fourth, the absence of triangulation with budget execution data, expenditure tracking, or stakeholder interviews constrains assessment of sustainability and institutional feasibility.

Furthermore, the analytical framework applied equal weighting across the five domains, an approach chosen to avoid imposing normative assumptions about the relative importance of specific components of social protection. However, alternative weighting schemes particularly those assigning greater importance to financial-risk protection or income-support mechanisms, could produce different comparative rankings. In addition, the use of a 0–3 ordinal scale, while transparent and replicable, limits granularity and may smooth meaningful variation between countries. Although a longer or weighted scale could increase sensitivity, it would also introduce greater subjectivity in document interpretation. These methodological choices reflect a trade-off between analytical precision and comparability across diverse policy contexts.

The analytical framework, grounded in health-system domains, primarily captures direct costs of care-seeking and health-system financial protection. It does not comprehensively assess broader indirect costs of illness such as income loss, unemployment, caregiving burden, or long-term livelihood disruption. As such, the study evaluates health-sector contributions to social protection rather than full-spectrum social protection in the welfare-state sense.

Lastly, the absence of formal monitoring systems for household-level economic support may have led to underestimation of existing cost-mitigation interventions. In several settings, social welfare services provide transport assistance for patients unable to return home after hospitalization, and differentiated service delivery models (e.g., Mobile ART, community ART groups, or decentralized drug distribution points) reduce indirect costs such as transport and lost income. However, where such interventions are not systematically monitored or embedded within national policy documents, they may not be fully captured within a document-based review. As such, the readiness scores may underestimate informal or operational mechanisms that partially mitigate household financial burden.

## Conclusion

This comparative policy analysis across Kenya, Malawi, Mozambique, Nigeria, and Zambia demonstrates that recognition of social protection within infectious-disease strategies is increasing, yet its institutionalization remains partial and uneven. While governance, coordination, and implementation frameworks show moderate-to-high readiness within the health sector, formal financial-risk protection mechanisms particularly catastrophic-cost monitoring and income-support measures, remain weak or absent. These findings suggest that current policy frameworks reflect health-sector preparedness to support social protection, rather than comprehensive economic protection in the broader welfare-state sense. Achieving Universal Health Coverage and eliminating catastrophic costs will therefore require not only stronger health-financing reforms, but deeper multisectoral integration with national social welfare systems to ensure that infectious-disease control efforts genuinely protect households from economic vulnerability.

## Data Availability

The original contributions presented in the study are included in the article/Supplementary material, further inquiries can be directed to the corresponding author.
